# The Effects of Age and Reproduction on the Lipidome of *Caenorhabditis elegans*

**DOI:** 10.1155/2019/5768953

**Published:** 2019-05-09

**Authors:** Qin-Li Wan, Zhong-Lin Yang, Xiao-Gang Zhou, Ai-Jun Ding, Yuan-Zhu Pu, Huai-Rong Luo, Gui-Sheng Wu

**Affiliations:** ^1^Key Laboratory for Aging and Regenerative Medicine, Department of Pharmacology, School of Pharmacy, Southwest Medical University, Luzhou, Sichuan 646000, China; ^2^State Key Laboratory of Phytochemistry and Plant Resources in West China, Yunnan Key Laboratory of Natural Medicinal Chemistry, Kunming Institute of Botany, Chinese Academy of Sciences, Kunming, Yunnan 650201, China; ^3^University of Chinese Academy of Sciences, Beijing 100039, China

## Abstract

Aging is a complex life process, and a unified view is that metabolism plays key roles in all biological processes. Here, we determined the lipidomic profile of *Caenorhabditis elegans* (*C. elegans*) using ultraperformance liquid chromatography high-resolution mass spectrometry (UPLC-HRMS). Using a nontargeted approach, we detected approximately 3000 species. Analysis of the lipid metabolic profiles at young adult and ten-day-old ages among wild-type N2, *glp-1* defective mutant, and double mutant *daf-16;glp-1* uncovered significant age-related differences in the total amount of phosphatidylcholines (PC), sphingomyelins (SM), ceramides (Cer), diglycerides (DG), and triglycerides (TG). In addition, the age-associated lipid profiles were characterized by ratio of polyunsaturated (PUFA) over monounsaturated (MUFA) lipid species. Lipid metabolism modulation plays an important role in reproduction-regulated aging; to identify the variations of lipid metabolites during germ line loss-induced longevity, we investigated the lipidomic profiles of long-lived *glp-1*/notch receptor mutants, which have reproductive deficiency when grown at nonpermissive temperature. The results showed that there was some age-related lipid variation, including TG 40:2, TG 40:1, and TG 41:1, which contributed to the long-life phenotype. The longevity of *glp-1* mutant was *daf-16*-dependent; the lipidome analysis of *daf-16;glp-1* double mutant revealed that the changes of some metabolites in the *glp-1* mutant were *daf-16*-dependent, while other metabolites displayed more complex epistatic patterns. We first conducted a comprehensive lipidome analysis to provide novel insights into the relationships between longevity and lipid metabolism regulated by germ line signals in *C. elegans*.

## 1. Introduction

Life expectancy in humans has dramatically increased worldwide [1]. Aging is often accompanied by diseases and disabilities, including cardiovascular diseases, neurodegenerative diseases, infectious diseases, and cancers [2, 3]. Thus, understanding the physiological characteristics of aging is of extreme importance to enable populations to grow old in good health and without disease.

Several pathways involved in aging mechanisms (e.g., mitochondrial health, DNA damage, proteostasis imbalance, systemic inflammation, epigenetic modifications, and nutrient-sensing pathways) have been associated with undesirable metabolic alterations [4, 5]. Without exception, metabolism changes also occur during reproduction-regulated aging. Using comprehensive nontargeted metabolomics, we previously demonstrated that aging is a process of metabolome remodeling and that long-lived germ line-less *glp-1* mutants regulated the levels of many age-variant metabolites to attenuate aging, including pyrimidine metabolism, purine metabolism, citric acid cycle, glycerophospholipid metabolism, and starch and sucrose metabolism [6]. These findings elucidated the mechanism of reproduction-regulated aging based on the metabolism of small molecules of endogenous water. Undoubtedly, the metabolism of non-water-soluble molecules is also involved in reproduction-regulated aging.

Previous studies have reported that sacrificing fertility to induce lifespan extension is evolutionarily conserved [7, 8]. In *C. elegans*, germ line removal not only lengthens lifespan but also increases fat accumulation, as observed using lipid-labeling dyes and gas chromatography (GC) [9, 10]. Notably, a reproductive deficiency leading to enhanced fat accumulation has been observed in many other organisms, including both invertebrates (e.g., drosophila) [11, 12] and vertebrates (e.g., mice, rats, and monkeys) [13–15].

In *C. elegans*, a RNA-Seq study demonstrated that upon GSC removal the genes associated with lipid metabolism were upregulated by DAF-16/FOXO3A and/or TCER-1/TCERG1 [16]. These genes included enzymes that initiate *de novo* fatty acid synthesis (including *pod*-*2* (encodes acetyl CoA carboxylase, ACC), *mlcd*-*1* (malonyl CoA decarboxylase 1, MLCD), and *fasn*-*1* (encodes fatty-acid synthase, FAS)) and the genes encoding diacylglycerol acyltransferase (DGAT) enzymes, which catalyze the final step in triglyceride (TAG) production (including *dgat-2*, *acs-22*, *mboa-2*, *Y53G8B.2*, and *K07B1.4*). In addition, in *glp*-*1* mutants, expression of desaturase enzymes, which catalyze the conversion of saturated fatty acids (SFAs) into unsaturated fatty acids (UFAs), was enhanced in a NHR-49/PPAR*α*- and NHR-80/HNF4-dependent manner, thereby increasing the monounsaturated fatty acid MUFA and reducing SFA levels. Furthermore, expression of *fat*-*6* and *fat*-*7*, encoding stearoyl-CoA 9-desaturase (SCD) enzymes, which transform stearic acid (SA, a SFA) to oleic acid (OA, a MUFA), was obviously altered in *glp-1* mutants. Additionally, in *glp-1* mutants, expression of genes involved in *β*-oxidation was upregulated in an NHR-49/PPAR*α*-dependent manner [17]. Moreover, following GSC loss, upregulation of the expression of the genes encoding lipase and lipase-like protein (i.e., *lipl-4*, orthologous to the human lipase LIPA) was mediated by DAF-16/FOXO3A, TCER-1/TCERG1, or SKN-1/NRF2 [16, 18, 19]. These studies illustrated that in germ line loss animals, lipid catabolism and anabolism are simultaneously elevated, and germ line loss animals show fat accumulation phenotypes. Expectedly, in *glp-1* mutants, lipid staining and gas chromatography/mass spectrometry (GC/MS) analyses displayed increased levels of TAG and some fatty acids, including C 16:0, C 16:1n7, C 18:0, C 18:1n7, and C 18:3 [9, 10, 16]. However, these studies only highlighted changes in the levels of overall lipids and fatty acids in *glp-1* mutants, barely elucidating the role of individual intact lipid molecules in the regulation of aging by reproduction. Therefore, the aim of the present study was to clarify the role of lipid metabolism in the regulation of aging by reproduction based on lipidomics to obtain intact lipid information and lipid metabolic profiles.

Lipidomics, a branch of metabolomics, refers to the characterization and analysis of the lipid complement of biological systems. Lipidomics can be achieved through comprehensive measurement of the lipid metabolic profiles based on analytical chemistry principles and technological tools, particularly mass spectrometry [20, 21]. In recent years, reflecting the development of MS, lipidomic technology has been widely used in many fields, such as organic biofluids with environmental, pathological, or toxicological stress [22–25]. Furthermore, the lipidome has been used in aging studies. For example, the Leiden Longevity Study demonstrated that specific lipids are associated with familial longevity [26]. Additional lipidomic analyses revealed that some lipids containing phosphatidylethanolamines, phosphatidylcholines, and sphingomyelins are associated with aging in humans [27]. Other studies have revealed that phospho/sphingolipids may represent putative markers and biological modulators of healthy aging in humans [28]. Furthermore, studies have illustrated that PC(O-34:1) and PC(O-34:3) are positively associated with longevity and negatively associated with diabetes [29, 30].

The objectives of the present study were to characterize the lipid metabolic profiles of *C. elegans* hermaphrodites during aging and identify germ line signals that regulate aging. Here, we assessed the lipid metabolic phenotype of whole *C. elegans* between two physiological ages among different mutants (e.g., wild type, *glp-1* mutants, and *daf-16;glp-1* double mutants) using ultraperformance liquid chromatography high-resolution mass spectrometry (UPLC-HRMS), with multivariate statistics analysis, including unsupervised principal component analysis (PCA) as well as hierarchical and supervised orthogonal projection to latent structure with discriminant analysis (OPLS-DA). These results demonstrated that reproductive signals affect lifespan by regulating metabolic changes, and some of these metabolic controls were mediated by FOXO/DAF-16.

## 2. Materials and Methods

### 2.1. Culture of Nematodes

Wild-type N2, CF1903 *glp-1(e2144)III*, and CF1880 *daf-16(mu86)I;glp-1(e2144)III* were obtained from the Caenorhabditis Genetics Center (CGC) and maintained under standard conditions on nematode growth media (NGM) with *Escherichia coli* OP50 as previously described, unless otherwise stated [31].

### 2.2. Sample Preparation for Metabolomic Analysis

The strains were cultured for 2-3 generations prior to collection. To eliminate germ cells of the *glp-1(e2144)* and *daf-16(mu86);glp-1(e2144)* alleles, all strains were synchronized and incubated at 20°C for 12 h, shifted to 25°C, grown to the L4 stage, and subsequently shifted back to 20°C, followed by harvest. The samples were prepared as previously described [6]. Briefly, a large sample of young adult (YA) or day-10 adult (10A) worms (~8000) was pooled and washed with M9 buffer and subsequently collected. All samples were snap frozen in liquid nitrogen and dried overnight *in vacuo* at a low temperature, weighed, and stored at -80°C until extraction. For samples of old worms, 10 *μ*M 5-fluoro-2′-deoxyuridine (FUdR, Sigma) was used to prevent self-fertilization. It is known that FUdR influences metabolism of worms [32], but the biomass of worms required for lipidomic analysis cannot be collected without using FUdR or a similar intervention. While in order to reduce the influence of FUdR, all strains were maintained on the same condition, as previously described [6].

Lipid metabolites from *C. elegans* samples were extracted three times with 600 *μ*L of precooled CH_2_Cl_2_/MeOH (2 : 1) using a TissueLyser at 55 Hz for 90 s. All extracts were subjected to centrifugation (12000 rpm for 10 min at 4°C), subsequently stored at -80°C until further analysis.

### 2.3. Lipidomic Analysis Using UPLC-HRMS

#### 2.3.1. Sample Preparation

The samples were thawed at room temperature, centrifuged at 12,000 rpm for 10 min at 4°C, and analyzed using UPLC-HRMS.

#### 2.3.2. Apparatus and Analytical Conditions

According to Witting et al. [33], liquid chromatography was performed using a reversed-phase C18 column (CORTECS UPLC C18, waters, 1.6 *μ*m 150 × 2.1 mm diameter column) with a flow rate of 300 *μ*L/min at 35°C, and 5 *μ*L of sample was injected. The mobile phase comprised ACN/H2O (3 : 2, *v*/*v*) (A) and iPrOH/ACN (9 : 1, *v*/*v*) (B), and both A and B contained 0.1% formic acid and 5 mM ammonium formate. The initial eluent comprised 32% solvent B, which was held for 2 min; the percent of buffer B was then gradually increased to 97% in 30 min, was held for 5 min, and was then returned to the initial condition in 0.1 min. The column was reequilibrated for 4.9 min, and the total run time was 40 min.

Analyses were conducted using the Agilent 1290 UPLC System (Agilent, Santa Clara, CA) connected to an Agilent 6500 Q-TOF Mass Spectrometer (Agilent, Santa Clara, CA). The parameter of mass spectrometry analyses in positive ion mode was set previously described, with some modifications [6].

#### 2.3.3. Data Processing

Raw files from UPLC-MS were converted to the mzData format using Masshunter Qualitative Software (Agilent, Santa Clara, CA). Peak detection, alignment, and integration were performed using the open-source software XCMS and CAMERA implemented with the freely available R statistical language (version 3.2.2). The procedures and parameters were conducted according to previous studies, with some modifications [34]. Identification of metabolites was performed based on their molecular ion masses and MS^n^ fragmentation compared with the literature and metabolomic library entries of purified standards, such as LIPID MAPS [35], LIPID BANK [36], the HMDB [37], Lipidhome [38], and LIPIDAT [39]. Subsequently, the putative identifications were verified through comparisons of the retention time matches to those of authentic standard compounds. In addition, we also purchased some commercially available purified standard compounds from Avanti Polar Lipids (Alabaster, AL, USA) to identify metabolites. These standard compounds include heptadecasphing-4-enine, heptadecasphinganine, C17 Sphingosine-1-phosphate, C17 Sphinganine-1-phosphate, SM(d18:1/12:0), Cer(d18:1/12:0), CerP(d18:1/12:0), Cer(d18:1/25:0), d5-DG(14:0/0:0/14:0), d5-DG(15:0/0:0/15:0), d5-DG(16:0/0:0/16:0), d5-DG(17:0/0:0/17:0), d5-DG(19:0/0:0/19:0), d5-TG(20:0/20:1/20:0), d5-TG(20:2/18:3/20:2), d5-TG(20:4/18:2/20:4), d5-DG(20:5/0:0/20:5), d5-TG(14:0/16:1/14:0), d5-TG(20:5/22:6/20:5), d5-TG(15:0/18:1/15:0), d5-TG(16:0/18:0/16:0), d5-TG(17:0/17:1/17:0), d5-TG(19:0/12:0/19:0), d5-TG(20:0/20:1/20:0), d5-TG(20:2/18:3/20:2), d5-TG(20:4/18:2/20:4), PE(17:0/14:1), and PC(17:0/14:1).

### 2.4. Bioinformatics and Statistical Analyses

Nontargeted lipidomic analyses were performed as previously described, with some modifications [6]. For the UPLC-MS data, after normalizing against the dry weights, the resolved data sets were subjected to statistical analysis. The algorithm for significance analysis of microarray (SAM) data (the false discovery rate FDR ≤ 0.05 unless otherwise noted) and unsupervised hierarchical clustering was performed using the web-based metabolomic data processing tool metaboAnalyst [40]. PCA and OPLS-DA were performed using SIMCA-P11.5. Other statistical calculations were conducted using PASW Statistics 20 (SPSS, Chicago, USA), and a *p* value of 0.05 or less was considered significant in every comparison.

MUFA-to-PUFA ratios were calculated as previously described [26]. Briefly, adding levels of all MUFA lipids (lipids with one double bond in any acyl chain), resulting value was divided by the sum of all PUFA lipids (species with three or more double bonds in their acyl chains).

## 3. Results

### 3.1. Lipidomic Profile Associated with Aging in *C. elegans*

**Figure 1:**
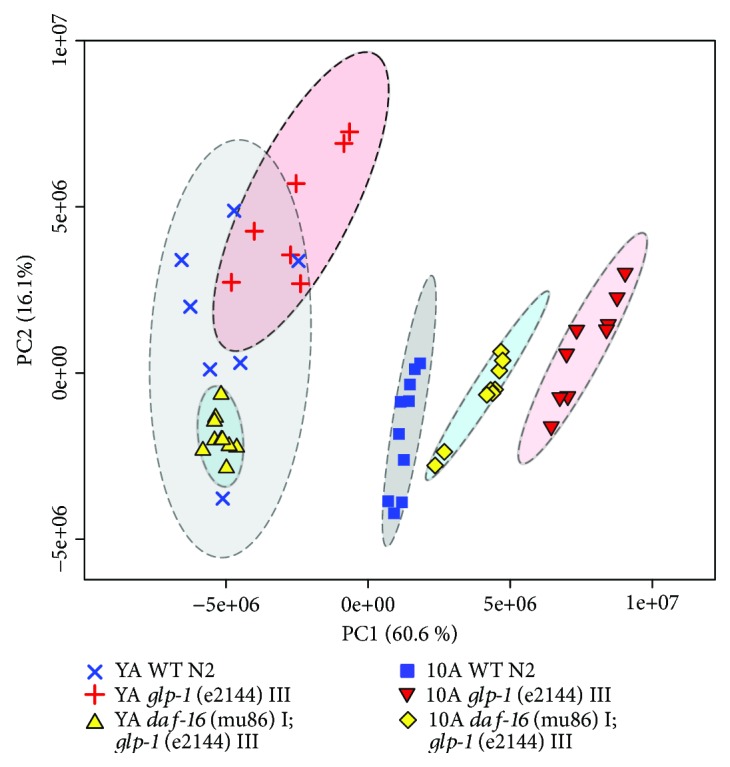
Lipid metabolic variations in WT, *glp-1(e2142)*, and *daf-16(mu86);glp-1(e2141)* worms. PCA included young adults and 10-day adults WT, *glp-1*, and *daf-16;glp-1*. PC1 and PC2 represent the first and second principal components, respectively.

**Figure 2:**
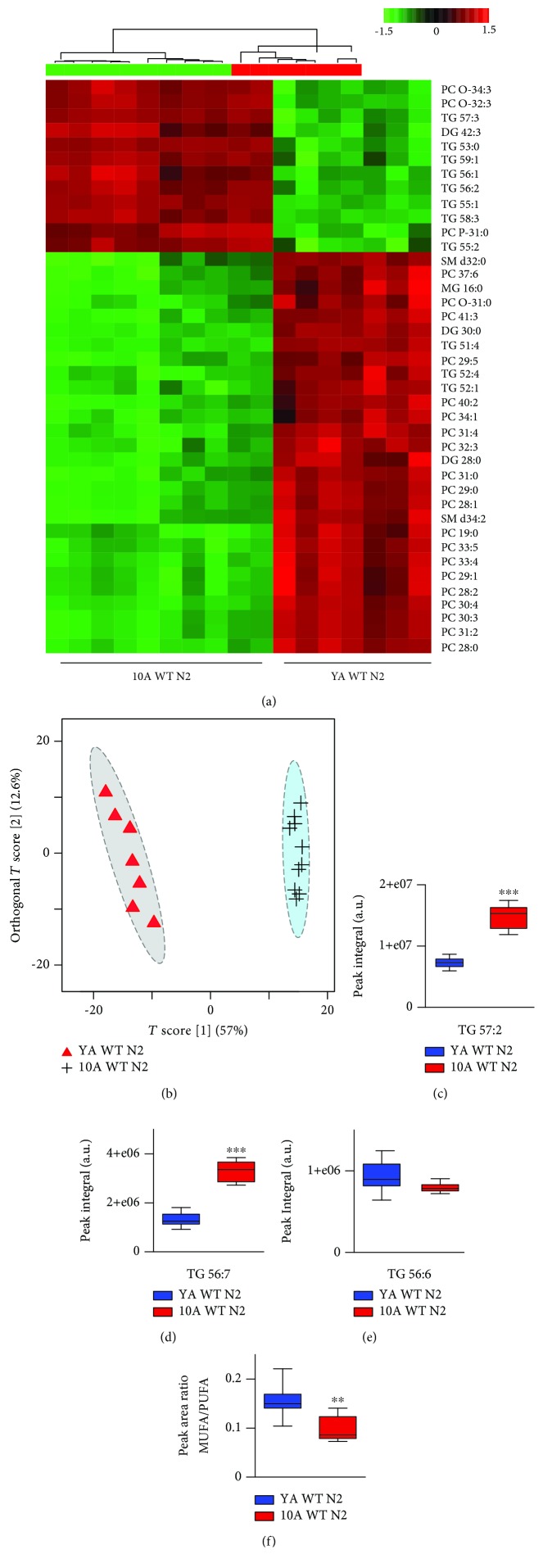
Age-related comprehensive lipidomic analysis in wild-type *C. elegans* (a) lipidomic analysis for YA (young adult) and 10A (10 days of adulthood) wild-type N2. Heat map plot showing the 40 most important different metabolites from the samples according to their aging status. Data are presented using hierarchical clustering (Pearson's correlation coefficient). Metabolite abundance levels were reflected in the heat maps using colors, with blue representing lower and red representing higher levels when comparing the mean metabolite abundance values. The distance function 1-correlation was used in hierarchical clustering to determine the order of metabolites and animals. (b) Scores from OPLS-DA model for YA and 10A wild-type N2. The peak integral (a.u.) of (c) TG 57:2, (d) TG 56:7, and (e) TG 56:6 in YA N2 and 10A N2. (f) MUFA-to-PUFA ratio differences in YA N2 and 10A N2. The *p* values were calculated using the Mann-Whitney *U* test, and 0.05 or less was considered significant. All statistical analyses were conducted using SPSS package.

The first aim of the present study was to characterize changes in the lipidomic profile during aging in *C. elegans* to identify novel lipids or groups of lipids as biomarkers of aging. To this end, we acquired the nontargeted UPLC-HRMS lipidomic profiles of wild-type N2 in young adults (YA) (egg laying has not commenced) and day 10 adults (10A) (aging, egg laying has ceased). Selecting these two time points diminished interference from the egg laying and stochastic components of aging in *C. elegans.* Using these samples (approximately 3000 molecules), multivariate statistical analyses were performed, including unsupervised PCA [41] and supervised OPLS-DA [42] models. PCA and OPLS-DA score plots showed precise separation when the two groups were compared (Figures 1 and 2(b)). In addition, detailed lipid analysis suggested that in old worms the concentrations of DG, PC, and SM significantly decreased, while that of Cer increased (Figure S1). These results suggested that aging included the reprogramming of lipid metabolism.

### 3.2. Identification of a Lipid Set with respect to Age

We displayed the top 40 different lipids for 10A worms compared with YA worms using heat mapping. The resulting heat map displayed a clear variation in the concentrations of metabolites with respect to age. Subsequently, to assess the individual discriminant of each component of the signature, *t*-test (2-tailed) or Mann-Whitney *U* tests basing on checking for normal distribution were performed (data not shown).

Compared with YA worms, the concentration of long-chain glycerol lipids (such as TG 57:3, TG 59:1, TG 58:3, and DG 42:3) increased in 10A worms, while the concentration of short-chain glycerol lipids (such as DG 30:0, TG 51:4, and DG 28:0) decreased. Additionally, glycerophospholipid levels changed for selected species with advanced age, and the concentration of most PCs was decreased in aged worms (such as PC 29:5, PC 34:1, PC 29:1, and PC 28:2), and the concentrations of SM 32:0 and SM 34:2 were also decreased in aged animals (Figure 2(a)). In addition, consistent with a previous study on the lipidomics of familial longevity, these results showed that the concentrations of TG 57:2 and TG 56:7 significantly increased in aged animals (Figures 2(c) and 2(d)), although TG 56:6 did not (Figure 2(e)) [26].

Content differences in polyunsaturated (PUFA) and monounsaturated (MUFA) lipids determine membrane peroxidation, and the MUFA-to-PUFA ratio has been suggested to be a marker of longevity [43, 44]. Therefore, we determined differences in the MUFA-to-PUFA ratio between YA and 10A worms. 10A worms displayed a lower MUFA-to-PUFA ratio compared with young worms (Figure 2(f)).

### 3.3. Lipidomic Characterization of Long-Lived *glp-1* Mutants according to Longevity

**Figure 3:**
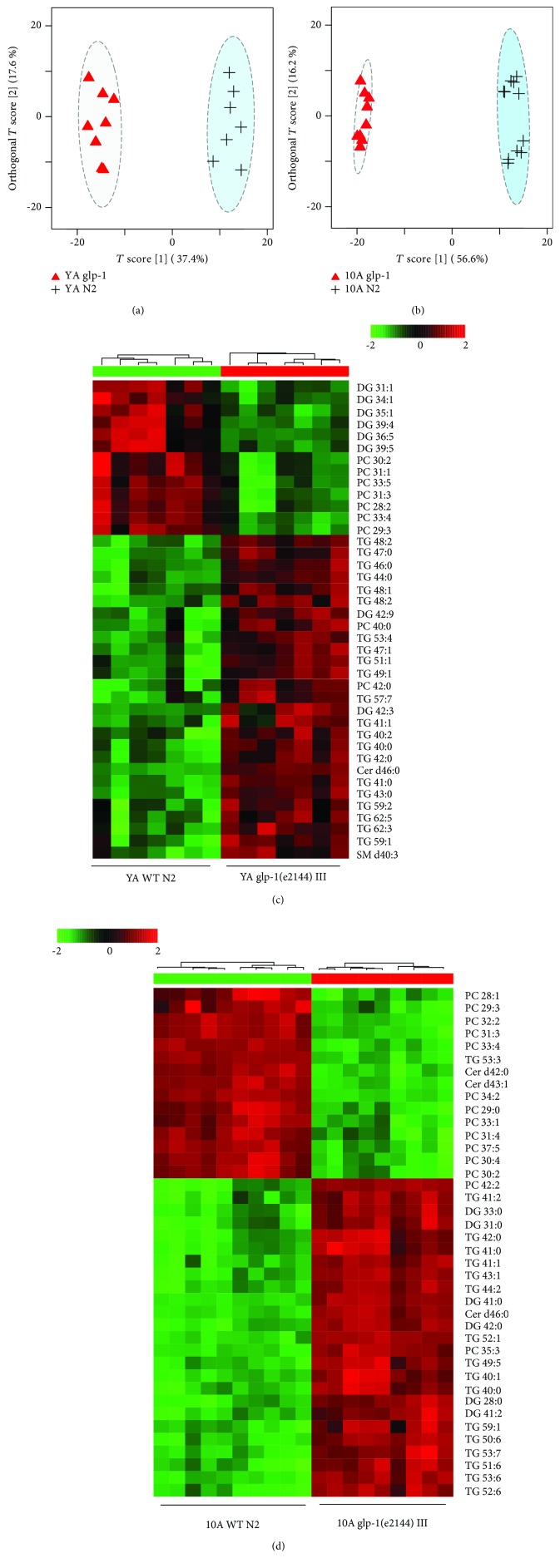
The long-lived *glp-1(e2144)* mutant has a distinctive lipid metabolic profile. (a) Scores from the OPLS-DA model for *glp-1* and wild-type N2 at (a) young adult stage and (b) 10 days of adulthood. Heat map plot showed the top 40 most important different metabolites from *glp-1* against N2 at (c) young adult stage and (d) 10 days of adulthood.

Germ line elimination, leading to lifespan extension in *C. elegans*, is successfully simulated by mutations that cause GSC loss and sterility. One such temperature-sensitive mutant, *glp-1(e2144ts)*, has been widely used as a model for lifespan extension resulting from germ line elimination. Fat metabolism, reproduction, and aging are intertwined regulatory axes, and previous studies have demonstrated that the longevity phenotype of the *glp-1* mutant is associated with the regulation of lipid metabolism and lipid homeostasis using fat staining and GC to detect the fat content [10, 16, 18]. However, the specific lipids regulated by *glp-1* to extend lifespan remain unknown. To demonstrate the relationship between lipid metabolism and aging in the *glp-1* mutant, we detected the intact lipid profiles in the *glp-1* mutant using a comprehensive lipidomic analysis method. Unsupervised PCA analysis showed that the *glp-1* mutant and wild-type samples have distinct lipid metabolic profiles at either the YA or 10A stage, albeit with little overlap at the young adult stage, which also indicated that aging increases metabolic differences (Figure 1). Further supervised OPLS-DA analysis revealed dramatic changes in the lipid metabolism of the *glp-1* mutant compared with the wild type at either the YA or 10A stage (Figures 3(a) and 3(b)).

Furthermore, the top 40 significantly different lipids of the YA and 10A *glp-1* mutants compared with wild-type worms were identified using heat mapping, and the results are listed in Figures 3(c) and 3(d). In addition, fat alterations associated with longevity primarily reflected the accumulation of short-chain lipids (such as TG 40:2, TG 40:1, and TG 41:1), which decreased in aged wild-type worms compared with young worms (Figures 3(c) and 3(d)). These finding suggested that the *glp-1* mutant regulated lipid metabolism to younger profiles compared with wild type.

**Figure 4:**
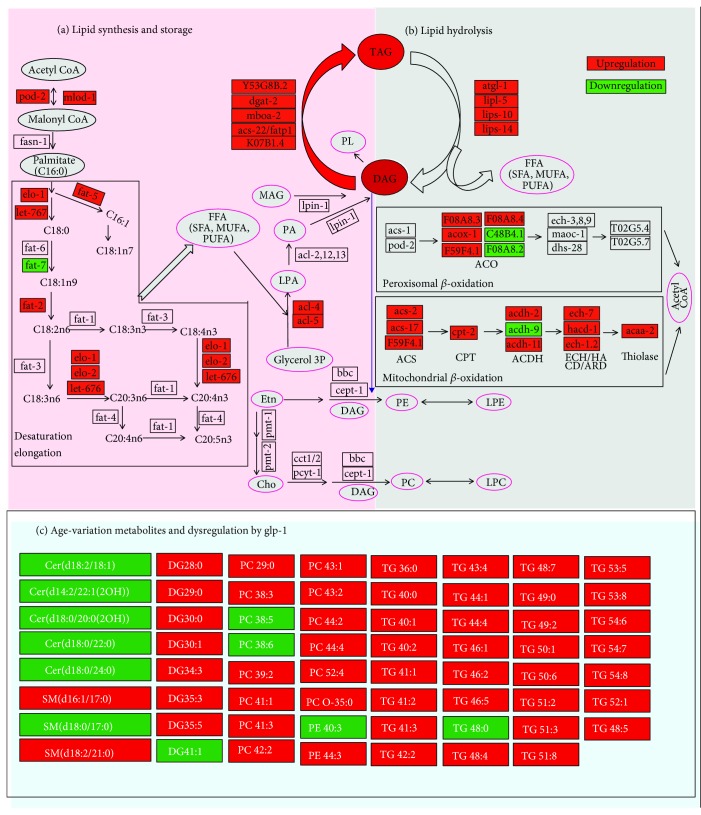
*glp-1* regulated age-variation lipid to achieve the longevity phenotype. (a, b) *glp-1* regulated the key genes involved in lipid anabolism and catabolism, which was modified from Amrit et al., *PLoS Genetics* 2016 [16]. (c) *glp-1* regulated age-variation lipid, and the colors reflect lipid changes, with yellow representing lower and red representing higher values when comparing *glp-1* mutants vs. N2.

**Figure 5:**
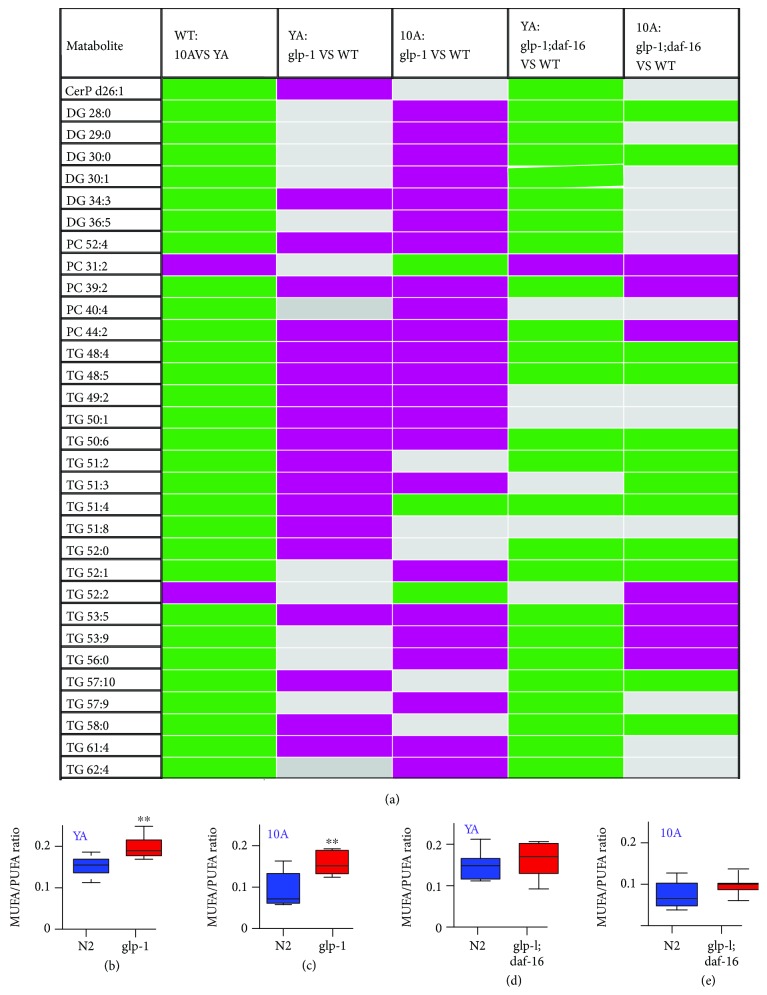
The age-related metabolic variation regulated by *glp-1* partly depends on FOXO/DAF-16. (a) Metabolite variation with age in *glp-1(e2144)* and *daf-16(mu86);glp-1(e2144)* double mutants in young adults and 10-day-old adults. Metabolite abundance levels were reflected using colors, increase (green) or decrease (purple) in metabolite concentrations, gray represents no significant difference, when mutants vs. N2. MUFA-to-PUFA ratio differences when *glp-1* vs. N2 at (b) young adult stage or (c) 10-day-old adult stage, or when *daf-16;glp-1* vs. N2 at (d) young adult stage or (e) 10-day-old adult. *p* values that were calculated using the Mann-Whitney *U* test of 0.05 or less was considered significant. All statistical analyses were calculated using SPSS package. *p* values were calculated using the Mann-Whitney *U* test, and the *p* value of 0.05 or less was considered significant. All statistical analyses were calculated using SPSS package.

Additionally, we used RF™ and univariate analyses to identify biomarkers associated with the long-lived phenotype and observed that *glp-1* dysregulated some age-variation lipids (such as PC 44:2, TG 48:4, and TG 50:1) to extend lifespan (Figure 4(c)), although some lipids were not changed (such as DG 34:1, DG 42:3, PC 33:4, and TG 60:3). These results indicated that not all aspects of aging were reset in long-living *glp-1* mutants. More detailed information regarding the significant metabolite changes in *glp-1* mutants compared with wild-type worms are listed in the supplemental information, Tables S1 and S2. In addition, the age-variation MUFA-to-PUFA ratio was obviously increased in *glp-1* mutants compared with wild type at either the YA or 10A stage (Figures 5(b) and 5(c)).

### 3.4. FOXO/DAF-16 Mediate Lipid Metabolism Reprogramming in *glp-1* Mutants

Previous studies have demonstrated that the transcription regulators DAF-16/FOXO are essential for the longevity of germ line-less adults, enhancing the expression of multiple genes involved in lipid metabolism and gene classes that promote longevity [16, 18]. Here, we used lipidomic analysis to reveal how germ line loss mutants depend on DAF-16 to regulate lipid metabolism to achieve the longevity phenotype. The results of multivariate analysis, including PCA and OPLS-DA, revealed that the lipid metabolism profiles of *daf-16;glp-1* mutants were different from those of wild type and the *glp-1* mutant (data not shown). Further univariate analysis indicated that some age-related metabolites regulated by germ line loss signals were *daf-16*-dependent (such as DG 34:3; TG 49:2, and TG 51:3) (Figure 5(a)), while other metabolites showed more complex epistatic patterns, such as TG 46:5, TG 40:1, and PC 38:3. More detailed information is provided in the supplemental information, Tables S1 and S2. In addition, the age-related MUFA-to-PUFA ratio was upregulated in the *glp-1* mutant, while no variation was observed in the *daf-16;glp-1* mutant, which may indicate that the age-related MUFA-to-PUFA ratio regulation in *glp-1* was mediated by *daf-16* (Figures 5(b), 5(c)–5(e)).

## 4. Discussion

Untargeted lipidomics enables the comprehensive investigation of endogenous lipids in complex biological systems and has the potential to identify modifications in metabolic pathways and networks in response to biological effects [45, 46]. The role of lipids in aging diseases and human longevity has been widely acknowledged, and the metabolic changes of several lipids associated with age-related diseases, such as hypertension, diabetes type 2, and cardiovascular disease, have been identified [47–49]. Therefore, we investigated the aging-related lipid metabolic variation in *C. elegans*. Analysis of the age-variation lipid profiles in aged worms revealed that the levels of DG, PC, and SM were significantly decreased, while that of Cer was increased. In addition, these results showed that accumulation of long-chain lipid and deleterious short-chain lipids in aged worms might contribute to aging. The aging-related lipid profile was also characterized by a lower MUFA-to-PUFA ratio. The present study is the first to report the age-related lipidomic profiles in *C. elegans*. The lipid profile, representative of healthy aging in *C. elegans*, comprised higher levels of ether PC and SM species and lower levels of PE and long-chain TG species.

In the present study, we observed that the levels of some SM significantly decreased, while those of Cer increased in aged worms compared with young animals. Sphingomyelin species are important components of tissue membranes and cellular messengers and are particularly abundant in neuronal cells. Low levels of SM species have been associated with aging-related diseases, including Alzheimer's, Parkinson's, Huntington's [50], diabetes [30], subclinical atherosclerosis [51], and cardiovascular disease [52]. Sphingomyelinase (SMase) is an enzyme that hydrolyzes SM species into ceramides, whose activity tends to increase with age, thereby decreasing SM levels and increasing ceramide levels [53, 54]. In addition, consistent with the results of the present study, a recent lipidomic study of familial longevity and aging kidney uncovered that the levels of SM species decreased in aged individuals [26, 27]. Thus, alterations in ceramide metabolism may directly influence aging.

We also detected decreasing levels of PC species. PC species prevent the oxidation of polyunsaturated fatty acids in lipoproteins [55]. In addition, upregulation of ether phospholipids is associated with a lower risk for hypertension, diabetes, and aging kidney [27, 30]. In addition, consistent with the results of the present study, a familial longevity study revealed that a higher level of PC represents a younger lipid profile [26]. Thus, PC species were present at lower levels in aged worms, consistent with the decreased antioxidant capacity of these molecules.

Long-chain triglycerides undergo beta-oxidation or peroxisomes, whose enzymatic capacity decreases with age [26]. Therefore, in aged worms, higher levels of long-chain TG and lower level of short-chain TG may reflect a declined beta-oxidation function compared to younger worms.

The fact that the MUFA-to-PUFA ratio is a longevity biomarker is consistent with reports showing a negative correlation between the membrane double bond content and longevity in mammals [56, 57]. SFA and MUFA are more essentially resistant to peroxidation than PUFA; thus, higher PUFA levels may increase lipid peroxidation and oxidative damage [56, 58]. Thus, in aged worms, a lower MUFA-to-PUFA ratio may be more prone to lipid peroxidation and oxidative damage. Altogether, the lipidome of aged worms, with a lower PC and higher polyunsaturated TG species, may reflect the accumulation of oxidative damage in aged animals.

**Figure 6:**
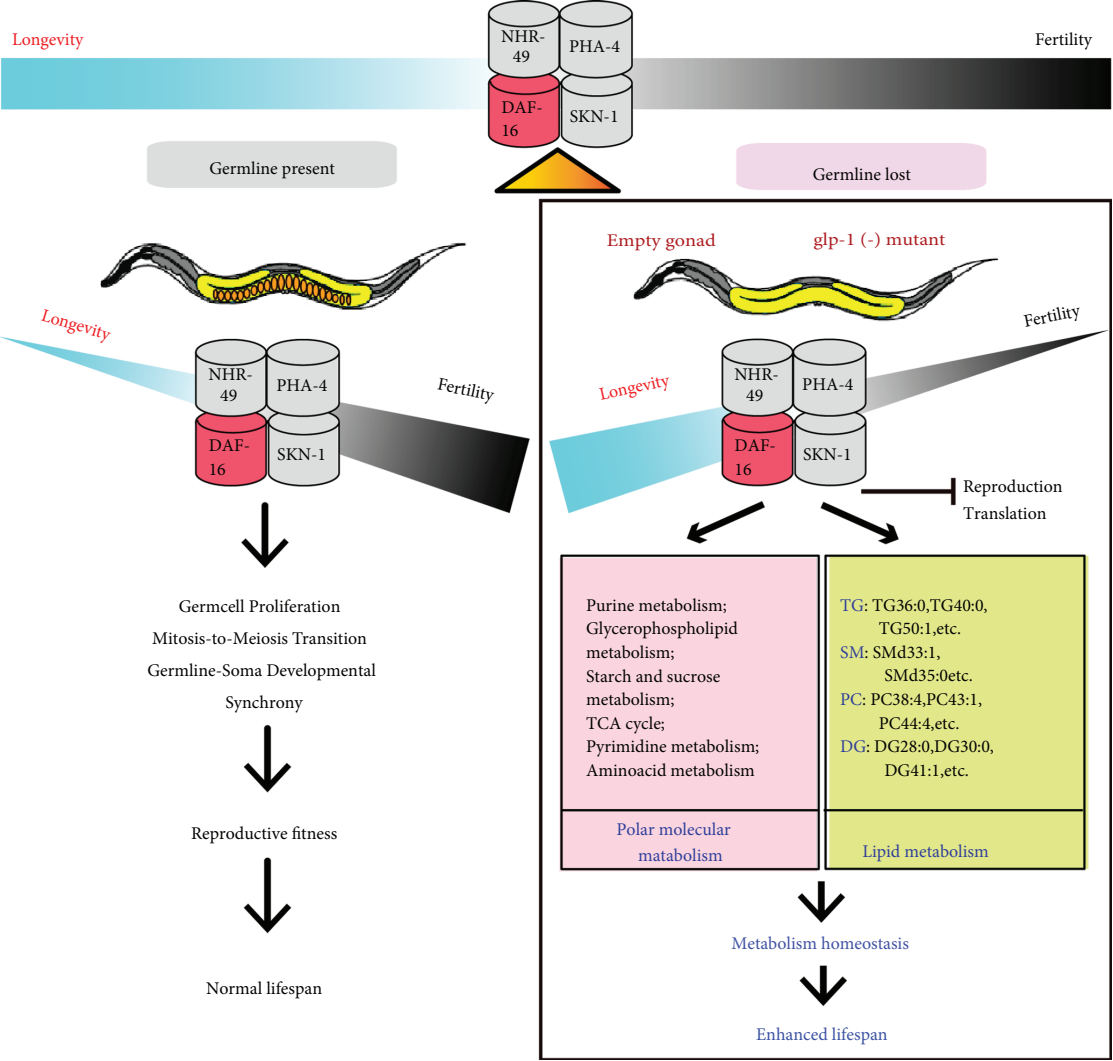
Metabolomic and lipidomic signatures with reproduction-regulated aging in *Caenorhabditis elegans.*

A previous study reported that *glp-1* mutants showed increased fat storage at the nonpermissive temperature relative to the wild type (N2) by using oil red O staining [16]. Other transcriptomic studies revealed that many aspects of lipid metabolism were altered in *glp-1* mutants, including fatty acid (FA) oxidation, FA desaturation, and triglyceride lipase [16, 18]. Another study showed that some FA (i.e., C16:0, C16:1n7, C18:0, and C18:3) changed in *glp-1* mutant through GC analysis [16, 18]. However, these studies did not determine which intact lipids were regulated by *glp-1* to achieve lifespan extension (Figures 4(a) and 4(b)), although total fatty acid analysis can provide insight into fatty acid metabolism. Extending upon previous studies, we analyzed the lipidome of *glp-1* mutants. Consistent with the results of a previous study, these results demonstrated that fat storage elevated in *glp-1* mutants. Compared to previous studies, we observed more special intact lipid molecule information regulated by *glp-1*. We observed that *glp-1* regulated some age-related lipids to extend lifespan, including PC 44:2, TG 48:4, and TG50:1 (Figures 4(c) and 5(a)). Furthermore, analysis of the lipidomics of the *daf-16;glp-1* double mutant revealed that DAF-16 partially mediated *glp-1*-regulated age-variation lipid metabolism. Indeed, a previous study reported that other downstream target genes also mediated *glp-1* to regulate lipid metabolism to extend lifespan, such as SKN-1 [18], TCER-1 [16], and NHR-49 [17]. Combined with the results of a previous metabolomic studies, there was an inseparable relationship between the extension of the lifespan of *glp-1* and aging-related metabolic changes (Figure 6).

To our knowledge, the present study is the first to use a comprehensive high-resolution lipidome to reveal the relationship between longevity and lipid metabolism. While there are some drawbacks of the lipidomic method, we obtained putative matches for lipids based on databases and references, but these matches often carry considerable uncertainty. In some cases, a single mass-charge ratio may match more than 10 putative known molecules. In addition, some lipids (such as TG (18:1/18:1/18:0) and TGs (18:0/18:2/18:0)) share the same molecular formulas and molecular weights. Thus, the only difference between these molecules is the position of the double bond. These molecules are typically considered to be TGs (54:2). Thus, it is difficult to obtain accurate lipid structure information from lipidomic studies. Moreover, it is difficult to conduct lipid metabolic set enrichment analysis, reflecting the relatively few known lipid metabolic pathways. Therefore, the present study likely represents only a variation of individual lipid molecules during aging but not the lipid metabolic pathway.

Nevertheless, the results of the present study show the power of using lipidomics to better understand the phenotype of aging and longevity in *C. elegans.* In future studies of aging mechanisms, this knowledge could be linked to functional studies. In any case, the findings of the present study, consistent with the available evidence, clearly suggest that lipid metabolism is closely associated with the aging process and that the long-lived phenotype of *glp-1* is closely related to the regulation of lipid metabolism.

## Data Availability

All the figures and tables used to support the findings of this study are included within the article and supplementary materials.

## Supplementary Materials

Supplementary Materials Figure S1: heat map plot listed the lipid variation between YA and 10A N2. Table S1: list of altered lipids of each mutant strain against the wild type in young adults. Table S2: list of altered lipids of each mutant strain against the wild-type days 10 of adult worms.
