# Supplementary material for: The Effects of Age and Reproduction on the Lipidome of Caenorhabditis elegans
Source: Oxid Med Cell Longev. 2019 May 9;2019:5768953. doi: 10.1155/2019/5768953 (PMC6532275; doi:10.1155/2019/5768953)
Supplement: Supplementary Materials — Figure S1: heat map plot listed the lipid variation between YA and 10A N2. Table S1: list of altered lipids of each mutant strain against the wild type in young adults. Table S2: list of altered lipids of each mutant strain against the wild-type days 10 of adult worms. [file 5768953.f1.docx]

**The Effects of Age and Reproduction on the Lipidome of *Caenorhabditis elegans***

Qin-Li Wan ^a^, Zhong-Lin Yang ^b, c^, Xiao-Gang Zhou ^a^, Ai-Jun Ding ^a^, Yuan-Zhu Pu ^a^, Huai-Rong Luo ^a, b^, Gui-Sheng Wu ^a^ *

^a^ Key Laboratory for Aging and Regenerative Medicine, Department of Pharmacology, School of Pharmacy, Southwest Medical University, Luzhou, Sichuan 646000, China

^b^ State Key Laboratory of Phytochemistry and Plant Resources in West China, Yunnan Key Laboratory of Natural Medicinal Chemistry, Kunming Institute of Botany, Chinese Academy of Sciences, Kunming, Yunnan 650201, China

^c^ University of Chinese Academy of Sciences, Beijing 100039, China

* Corresponding author: Dr. Gui‐Sheng Wu

Key Laboratory for Aging and Regenerative Medicine, Department of Pharmacology

School of Pharmacy, Southwest Medical University

319 Zhongshan Road, Luzhou, Sichuan 646000, China

Phone: +86 830-3160842; Fax: +86 830-3160842

E-mail address: wgs@swmu.edu.cn

**Supplementary information**

**Contents:**

Fig. S1 Heatmap plot listed the lipid variation between YA and 10A N2.

Table S1 List of altered lipids of each mutant strain against the wild type in young adults.

Table S2 List of altered lipids of each mutant strain against the wild type days 10 of adult worms.

**Fig. S1**


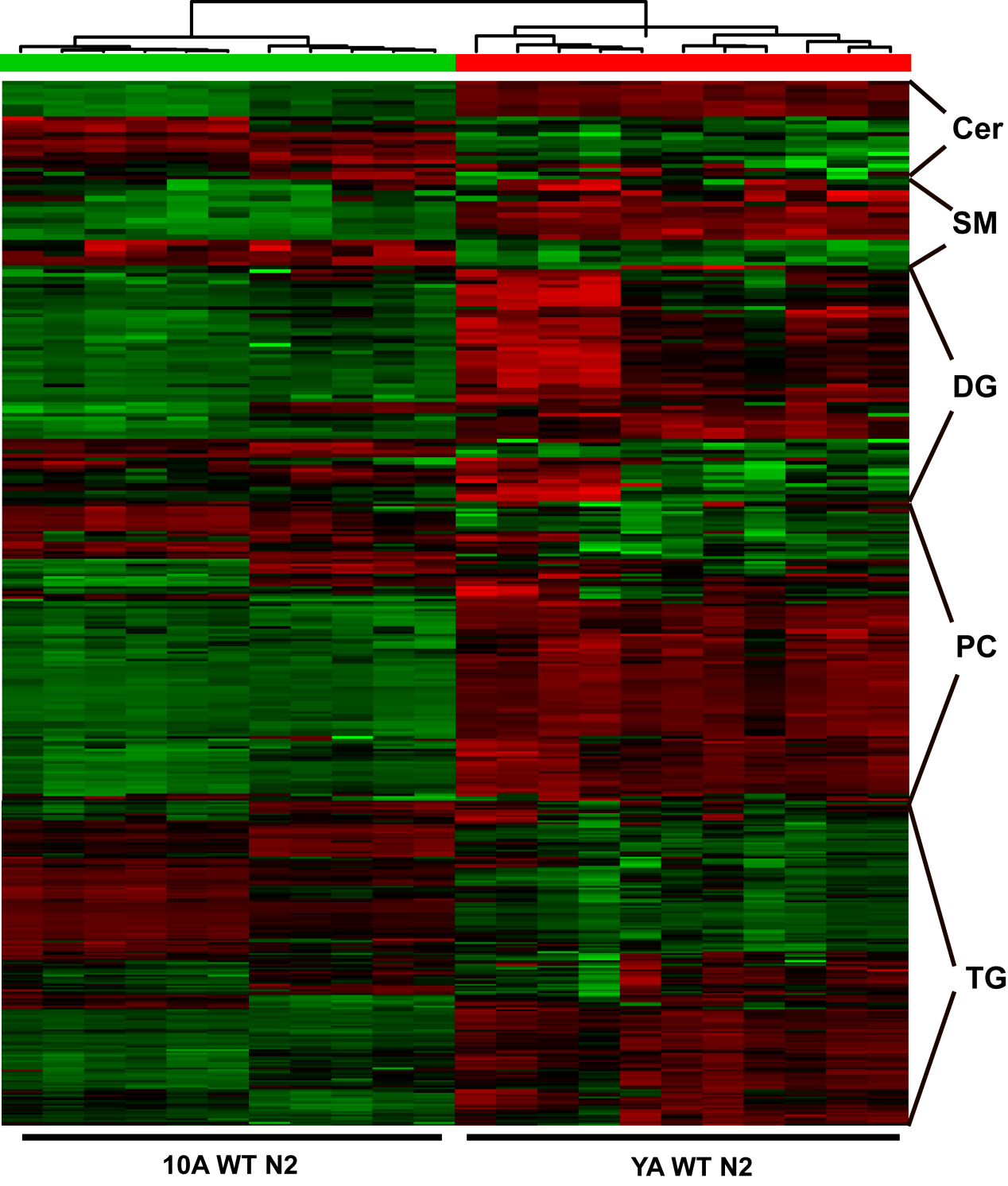


**Fig. S1** Heatmap plot listed the lipid variation between YA and 10A N2.

**Table S1**

| metabolites ( young adult) | *P* value  *glp-1* VS N2 | | *P* value  *daf-16;glp-1* VS N2 |
| --- | --- | --- | --- |
| Cer d36:1 (2OH) | 0.0016 | 0.2100 | |
| Cer d32:3 (2OH) | 0.0020 | 0.0798 | |
| Cer d34:3 (2OH) | 0.0422 | 0.0159 | |
| Cer d38:0 (2OH) | 0.0048 | 0.1909 | |
| Cer d42:0 | 0.0257 | 0.6044 | |
| Cer d37:1 | 0.0003 | 0.0965 | |
| Cer d40:1 | 0.3914 | 0.0006 | |
| Cer d46:0 | 0.0011 | 0.0005 | |
| Cer d43:1 | 0.6481 | 0.0313 | |
| Cer d44:1 | <0.0001 | <0.0001 | |
| DG 28:0 | 0.0647 | 0.0021 | |
| DG 29:0 | 0.0727 | 0.0008 | |
| DG 30:0 | 0.0595 | 0.0004 | |
| DG 30:1 | 0.0556 | 0.0026 | |
| DG 31:0 | 0.0825 | <0.0001 | |
| DG 31:1 | 0.0028 | 0.0007 | |
| DG 32:1 | 0.0822 | 0.0016 | |
| DG 32:2 | 0.5386 | 0.0111 | |
| DG 33:0 | 0.023 | 0.0003 | |
| DG 33:2 | 0.0349 | 0.0083 | |
| DG 33:3 | 0.0023 | 0.4259 | |
| DG 34:1 | 0.0064 | 0.0012 | |
| DG 34:2 | <0.0001 | 0.1427 | |
| DG 34:3 | 0.0127 | 0.0052 | |
| DG 35:1 | 0.0001 | 0.0008 | |
| DG 35:2 | 0.0042 | 0.0460 | |
| DG 35:4 | 0.0034 | 0.0569 | |
| DG 35:5 | 0.0028 | 0.8923 | |
| DG 36:1 | 0.0056 | 0.0677 | |
| DG 36:4 | 0.0092 | 0.0325 | |
| DG 36:5 | 0.161 | 0.0045 | |
| DG 37:0 | 0.0005 | 0.1043 | |
| DG 37:3 | 0.0039 | 0.2949 | |
| DG 37:4 | 0.0024 | 0.3045 | |
| DG 37:6 | 0.0020 | 0.6113 | |
| DG 38:2 | 0.0002 | 0.1846 | |
| DG 38:4 | 0.0072 | 0.1588 | |
| DG 38:5 | 0.0041 | 0.9831 | |
| DG 39:0 | 0.0006 | 0.0013 | |
| DG 39:3 | 0.0001 | 0.5700 | |
| DG 39:4 | <0.0001 | 0.0753 | |
| DG 39:5 | 0.0006 | 0.1306 | |
| DG 39:6 | 0.0018 | 0.8585 | |
| DG 40:1 | 0.0797 | <0.0001 | |
| DG 40:3 | 0.0032 | 0.0007 | |
| DG 40:5 | 0.0031 | 0.0047 | |
| DG 40:6 | 0.0019 | 0.3189 | |
| DG 40:7 | 0.0099 | 0.9463 | |
| DG 41:0 | 0.0049 | 0.0356 | |
| DG 41:1 | 0.0399 | <0.0001 | |
| DG 41:2 | 0.0003 | 0.2684 | |
| DG 42:3 | <0.0001 | 0.1254 | |
| PC 19:0 | 0.0129 | 0.0020 | |
| PC 25:0 | 0.0061 | <0.0001 | |
| PC 27:0 | 0.0013 | <0.0001 | |
| PC 28:0 | <0.0001 | 0.2431 | |
| PC 28:1 | <0.0001 | <0.0001 | |
| PC 28:2 | 0.0054 | <0.0001 | |
| PC 29:0 | <0.0001 | 0.00123 | |
| PC 29:1 | 0.0156 | <0.0001 | |
| PC 29:3 | 0.0002 | <0.0001 | |
| PC 30:1 | <0.0001 | <0.0001 | |
| PC 30:2 | 0.0047 | <0.0001 | |
| PC 30:3 | 0.2424 | 0.00758 | |
| PC 30:4 | 0.0009 | <0.0001 | |
| PC 31:0 | 0.0001 | <0.0001 | |
| PC 31:1 | 0.0001 | 0.00023 | |
| PC 31:2 | 0.7493 | 0.00135 | |
| PC 31:3 | 0.0008 | <0.0001 | |
| PC 31:4 | <0.0001 | 0.0005 | |
| PC 31:5 | <0.0001 | 0.0006 | |
| PC 32:2 | 0.3924 | 0.0009 | |
| PC 32:3 | 0.0018 | 0.0043 | |
| PC 32:4 | <0.0001 | 0.0008 | |
| PC 32:5 | 0.0013 | 0.7273 | |
| PC 33:0 | 0.0008 | <0.0001 | |
| PC 33:3 | 0.0051 | 0.0066 | |
| PC 33:4 | <0.0001 | 0.0005 | |
| PC 34:2 | 0.0086 | 0.0064 | |
| PC 34:3 | 0.0001 | 0.0005 | |
| PC 34:5 | 0.9300 | 0.0006 | |
| PC 35:0 | 0.2045 | 0.0038 | |
| PC 35:4 | 0.0021 | 0.1557 | |
| PC 35:6 | 0.0018 | 0.0610 | |
| PC 36:0 | 0.0376 | 0.3836 | |
| PC 36:1 | 0.9509 | 0.0067 | |
| PC 36:4 | <0.0001 | <0.0001 | |
| PC 36:5 | 0.0464 | 0.5119 | |
| PC 36:8 | 0.2688 | <0.0001 | |
| PC 37:0 | 0.8089 | 0.0002 | |
| PC 37:1 | 0.0002 | 0.0148 | |
| PC 37:6 | 0.0002 | <0.0001 | |
| PC 37:7 | 0.0027 | <0.0001 | |
| PC 38:0 | 0.0011 | 0.0332 | |
| PC 38:2 | 0.0001 | 0.0592 | |
| PC 38:3 | 0.003 | 0.0804 | |
| PC 38:5 | 0.139 | <0.0001 | |
| PC 38:6 | 0.0011 | 0.0169 | |
| PC 39:0 | 0.974 | 0.0028 | |
| PC 39:2 | 0.0240 | 0.0002 | |
| PC 39:5 | 0.0274 | 0.3087 | |
| PC 40:0 | 0.0011 | 0.814 | |
| PC 40:2 | 0.9183 | 0.0028 | |
| PC 40:3 | 0.0054 | 0.0016 | |
| PC 40:5 | 0.0566 | <0.0001 | |
| PC 41:3 | 0.0025 | 0.0305 | |
| PC 42:0 | 0.0189 | 0.0034 | |
| PC 42:4 | 0.1123 | 0.0063 | |
| PC 42:5 | <0.0001 | 0.0019 | |
| PC 43:0 | 0.0005 | 0.0049 | |
| PC 43:1 | 0.0174 | 0.0090 | |
| PC 44:2 | 0.0097 | 0.0059 | |
| PC 44:4 | 0.0448 | 0.0040 | |
| PC 44:5 | 0.5526 | 0.0018 | |
| PC 44:7 | 0.1557 | 0.0002 | |
| PC 46:2 | 0.2447 | 0.0011 | |
| PC 46:3 | 0.0089 | 0.1296 | |
| PC 46:4 | 0.0475 | 0.0007 | |
| PC 46:5 | 0.6676 | 0.0192 | |
| PC 46:6 | 0.1078 | 0.0012 | |
| PC 46:7 | 0.0567 | <0.0001 | |
| PC 48:2 | <0.0001 | 0.73542 | |
| PC 52:4 | 0.0005 | <0.0001 | |
| PC(O-16:0/19:0) | <0.0001 | 0.0189 | |
| PE(37:0) | <0.0001 | <0.0001 | |
| PE(40:0) | 0.5994 | 0.0015 | |
| PE(40:3) | 0.0066 | 0.9337 | |
| PE(42:0) | 0.0091 | 0.001 | |
| PE(44:3) | 0.0092 | 0.0122 | |
| PE(46:3) | 0.2429 | 0.0012 | |
| PE(46:4) | 0.4899 | <0.0001 | |
| PE(48:0) | 0.0055 | 0.0002 | |
| PE(48:1) | 0.0012 | 0.0042 | |
| PE(48:2) | 0.0024 | 0.7686 | |
| SM(d16:1/17:0) | <0.0001 | 0.3701 | |
| SM(d16:1/20:0) | 0.0055 | 0.1613 | |
| SM(d16:1/25:0) | 0.1811 | 0.0008 | |
| SM(d18:0/17:0) | 0.0013 | 0.6212 | |
| SM(d18:1/19:0) | 0.0181 | 0.0011 | |
| SM(d18:1/20:0) | 0.0017 | 0.5738 | |
| SM(d18:2/15:0) | 0.0092 | 0.4764 | |
| SM(d18:2/18:1) | 0.0323 | 0.0072 | |
| SM(d18:2/21:0) | 0.0557 | <0.0001 | |
| SM(d18:2/22:1) | <0.0001 | 0.0001 | |
| TG 40:0 | <0.0001 | <0.0001 | |
| TG 40:1 | 0.0131 | 0.01154 | |
| TG 40:2 | <0.0001 | <0.0001 | |
| TG 41:0 | <0.0001 | <0.0001 | |
| TG 41:1 | <0.0001 | 0.0033 | |
| TG 41:2 | <0.0001 | 0.0040 | |
| TG 41:3 | 0.0002 | 0.9911 | |
| TG 42:0 | <0.0001 | <0.0001 | |
| TG 43:0 | <0.0001 | <0.0001 | |
| TG 43:1 | 0.0099 | 0.5662 | |
| TG 43:4 | 0.0429 | 0.0528 | |
| TG 44:0 | <0.0001 | <0.0001 | |
| TG 44:4 | 0.0194 | 0.0054 | |
| TG 44:6 | 0.0840 | 0.0058 | |
| TG 45:0 | 0.0004 | <0.0001 | |
| TG 45:2 | 0.6106 | 0.0006 | |
| TG 45:4 | 0.1763 | 0.0003 | |
| TG 47:0 | 0.0023 | <0.0001 | |
| TG 47:1 | <0.0001 | <0.0001 | |
| TG 47:3 | 0.4723 | 0.0082 | |
| TG 47:5 | 0.2830 | <0.0001 | |
| TG 49:0 | 0.2053 | 0.0030 | |
| TG 49:1 | <0.0001 | <0.0001 | |
| TG 49:3 | 0.0030 | <0.0001 | |
| TG 49:4 | 0.247 | 0.0002 | |
| TG 51:1 | <0.0001 | <0.0001 | |
| TG 51:3 | 0.0059 | 0.5681 | |
| TG 51:4 | 0.0005 | <0.0001 | |
| TG 51:5 | 0.0043 | <0.0001 | |
| TG 51:6 | 0.8345 | <0.0001 | |
| TG 51:7 | 0.2131 | 0.0001 | |
| TG 51:8 | 0.0318 | 0.3563 | |
| TG 53:1 | 0.0041 | 0.0519 | |
| TG 53:2 | 0.0028 | 0.0679 | |
| TG 53:4 | <0.0001 | 0.0011 | |
| TG 53:5 | 0.0277 | <0.0001 | |
| TG 53:6 | 0.4716 | 0.0002 | |
| TG 53:7 | 0.2808 | <0.0001 | |
| TG 53:9 | 0.1993 | <0.0001 | |
| TG 55:1 | 0.0007 | 0.0003 | |
| TG 55:3 | 0.0011 | 0.0018 | |
| TG 55:4 | 0.0012 | 0.0038 | |
| TG 55:5 | 0.1804 | 0.0002 | |
| TG 55:6 | 0.0021 | <0.0001 | |
| TG 55:7 | <0.0001 | 0.0159 | |
| TG 55:8 | <0.0001 | 0.0024 | |
| TG 57:0 | 0.009 | 0.0525 | |
| TG 57:1 | 0.5401 | 0.0017 | |
| TG 57:11 | 0.0023 | 0.5842 | |
| TG 57:2 | 0.0016 | 0.0015 | |
| TG 57:3 | 0.3423 | 0.0083 | |
| TG 57:4 | 0.6318 | <0.0001 | |
| TG 57:5 | 0.6522 | <0.0001 | |
| TG 57:6 | 0.0011 | <0.0001 | |
| TG 57:7 | <0.0001 | 0.7106 | |
| TG 57:8 | 0.0067 | 0.5118 | |
| TG 57:9 | 0.5297 | 0.0005 | |
| TG 58:8 | 0.4325 | 0.0004 | |
| TG 59:3 | 0.0012 | 0.2764 | |
| TG 59:4 | 0.3003 | <0.0001 | |
| TG 59:6 | 0.1474 | 0.0003 | |
| TG 59:7 | 0.7624 | <0.0001 | |
| TG 61:3 | 0.0001 | 0.0087 | |
| TG 36:0 | <0.0001 | <0.0001 | |
| TG 42:2 | 0.0032 | 0.6197 | |
| TG 44:1 | 0.5518 | 0.0007 | |
| TG 44:2 | 0.0464 | 0.0035 | |
| TG 44:3 | 0.8292 | 0.0021 | |
| TG 45:1 | <0.0001 | 0.0006 | |
| TG 46:0 | 0.0008 | <0.0001 | |
| TG 46:1 | 0.0588 | 0.0014 | |
| TG 46:2 | 0.0053 | <0.0001 | |
| TG 46:3 | 0.9987 | <0.0001 | |
| TG 46:4 | 0.4396 | 0.0009 | |
| TG 46:5 | 0.0339 | <0.0001 | |
| TG 46:6 | 0.0033 | 0.0253 | |
| TG 47:2 | 0.7635 | <0.0001 | |
| TG 48:0 | 0.0320 | <0.0001 | |
| TG 48:1 | <0.0001 | <0.0001 | |
| TG 48:2 | <0.0001 | <0.0001 | |
| TG 48:3 | 0.4528 | 0.0033 | |
| TG 48:4 | 0.0361 | 0.0008 | |
| TG 48:5 | 0.0065 | <0.0001 | |
| TG 49:5 | 0.5755 | <0.0001 | |
| TG 50:0 | 0.0036 | <0.0001 | |
| TG 50:1 | 0.0460 | 0.2891 | |
| TG 50:2 | 0.0005 | 0.3765 | |
| TG 50:4 | 0.4761 | 0.0001 | |
| TG 50:5 | 0.4703 | 0.0024 | |
| TG 50:6 | 0.0040 | 0.0036 | |
| TG 52:0 | 0.0063 | 0.0015 | |
| TG 52:2 | 0.1283 | 0.3679 | |
| TG 52:5 | 0.1733 | <0.0001 | |
| TG 52:6 | 0.1305 | <0.0001 | |
| TG 52:8 | 0.5750 | 0.0001 | |
| TG 53:8 | 0.0036 | 0.0001 | |
| TG 54:1 | 0.0030 | 0.0021 | |
| TG 54:10 | 0.5609 | 0.0006 | |
| TG 54:2 | <0.0001 | 0.1128 | |
| TG 54:8 | 0.0142 | <0.0001 | |
| TG 54:9 | 0.3375 | <0.0001 | |
| TG 55:2 | 0.0001 | <0.0001 | |
| TG 56:0 | 0.4476 | 0.0032 | |
| TG 56:1 | 0.0029 | <0.0001 | |
| TG 56:2 | 0.0025 | 0.0002 | |
| TG 56:3 | 0.0001 | 0.0759 | |
| TG 56:4 | 0.0497 | 0.9932 | |
| TG 56:5 | 0.7742 | 0.1825 | |
| TG 56:6 | 0.0381 | <0.0001 | |
| TG 56:7 | 0.0011 | 0.0004 | |
| TG 56:8 | 0.0067 | <0.0001 | |
| TG 56:9 | 0.1644 | <0.0001 | |
| TG 58:1 | 0.0075 | 0.2298 | |
| TG 58:10 | 0.1729 | 0.0009 | |
| TG 58:3 | 0.0002 | 0.7335 | |
| TG 58:9 | 0.0821 | 0.0001 | |
| TG 59:1 | <0.0001 | 0.0009 | |
| TG 59:2 | <0.0001 | 0.0028 | |
| TG 60:1 | 0.0043 | 0.2624 | |
| TG 60:2 | 0.9764 | 0.0055 | |
| TG 60:3 | <0.0001 | <0.0001 | |
| TG 60:4 | 0.0082 | <0.0001 | |
| TG 60:5 | 0.5684 | <0.0001 | |
| TG 60:6 | <0.0001 | 0.0005 | |
| TG 60:7 | 0.0524 | 0.0051 | |
| TG 61:2 | 0.0033 | 0.2074 | |
| TG 61:4 | 0.0448 | 0.0026 | |
| TG 61:6 | <0.0001 | 0.0002 | |
| TG 62:1 | 0.0021 | 0.0678 | |
| TG 62:2 | 0.9538 | <0.0001 | |
| TG 62:3 | 0.0010 | 0.0045 | |
| TG 62:4 | 0.1904 | 0.0002 | |
| TG 62:5 | <0.0001 | 0.0162 | |
| TG 62:6 | <0.0001 | 0.1246 | |
| TG 62:7 | 0.0533 | 0.0006 | |
| TG 62:8 | 0.2971 | <0.0001 | |

**Table S1. List of altered lipid of each mutant strains against the wild type in young adults.** Metabolite abundance level were reflected using colors, and with yellow being lower and red higher when mutants VS. N2. *P* values were calculated by Mann-Whitney U test, and the *p*-value of 0.05 or less was considered significant. All statistical were calculated by using SPSS package**.**

**Table S2**

| metabolites (10-day-adult) | *P* value  *glp-1* VS N2 | *P* value  *daf-16;glp-1* VS N2 |
| --- | --- | --- |
| Cer d35:0 (OH) | <0.0001 | 0.3099 |
| Cer d36:1 (2OH) | 0.0010 | 0.0037 |
| Cer d32:2 (2OH) | 0.5614 | 0.0060 |
| Cer d34:3 (2OH) | 0.4417 | 0.0039 |
| Cer d36:3(2OH) | <0.0001 | 0.1025 |
| Cer d38:3 (2OH) | <0.0001 | 0.0118 |
| Cer d32:0 | <0.0001 | 0.6001 |
| Cer d33:0 | 0.0002 | <0.0001 |
| Cer d34:0 | <0.0001 | <0.0001 |
| Cer d34:1 | 0.9791 | 0.0035 |
| Cer d20:1 | 0.0038 | 0.0657 |
| Cer d41:1 | 0.9022 | <0.0001 |
| Cer d42:0 | 0.0258 | 0.5891 |
| Cer d43:1 | 0. 0359 | <0.0001 |
| Cer d46:0 | 0.0285 | 0.0646 |
| DG 28:0 | <0.0001 | <0.0001 |
| DG 29:0 | <0.0001 | 0.0524 |
| DG 30:0 | <0.0001 | <0.0001 |
| DG 30:1 | 0.0482 | 0.2696 |
| DG 31:0 | <0.0001 | <0.0001 |
| DG 31:2 | 0.0411 | 0.4855 |
| DG 32:0 | <0.0001 | 0.0003 |
| DG 33:0 | <0.0001 | 0.0008 |
| DG 33:2 | 0.1586 | 0.0010 |
| DG 33:3 | 0.0001 | 0.0006 |
| DG 34:0 | <0.0001 | 0.0055 |
| DG 34:1 | <0.0001 | 0.0660 |
| DG 34:2 | <0.0001 | <0.0001 |
| DG 35:0 | <0.0001 | 0.0007 |
| DG 35:1 | <0.0001 | 0.0012 |
| DG 35:2 | 0.0034 | 0.0034 |
| DG 35:3 | 0.0135 | 0.0343 |
| DG 35:5 | 0.8772 | <0.0001 |
| DG 35:6 | 0.0556 | <0.0001 |
| DG 36:1 | <0.0001 | <0.0001 |
| DG 36:3 | 0.2027 | 0.0195 |
| DG 36:4 | 0.0336 | 0.0015 |
| DG 37:0 | <0.0001 | <0.0001 |
| DG 37:2 | <0.0001 | 0.0016 |
| DG 37:3 | 0.0003 | 0.8758 |
| DG 37:4 | <0.0001 | 0.1929 |
| DG 38:2 | <0.0001 | 0.0081 |
| DG 38:5 | 0.0001 | <0.0001 |
| DG 39:0 | <0.0001 | <0.0001 |
| DG 39:3 | 0.0004 | 0.3598 |
| DG 39:4 | <0.0001 | 0.6177 |
| DG 40:1 | <0.0001 | <0.0001 |
| DG 40:3 | 0.0005 | 0.4599 |
| DG 40:6 | <0.0001 | 0.0065 |
| DG 41:2 | <0.0001 | <0.0001 |
| DG 42:0 | 0.0301 | <0.0001 |
| DG 42:3 | <0.0001 | 0.4659 |
| PC 19:0 | <0.0001 | <0.0001 |
| PC 25:0 | 0.1272 | 0.0011 |
| PC 28:0 | <0.0001 | <0.0001 |
| PC 28:1 | <0.0001 | 0.1853 |
| PC 29:3 | <0.0001 | 0.0229 |
| PC 30:2 | <0.0001 | 0.3652 |
| PC 30:4 | <0.0001 | 0.0105 |
| PC 31:0 | <0.0001 | 0.0004 |
| PC 31:1 | <0.0001 | 0.0272 |
| PC 31:2 | <0.0001 | 0.0005 |
| PC 31:3 | <0.0001 | 0.3241 |
| PC 31:4 | <0.0001 | 0.2885 |
| PC 31:5 | <0.0001 | 0.1474 |
| PC 31:6 | 0.3641 | 0.0045 |
| PC 32:0 | <0.0001 | <0.0001 |
| PC 32:1 | <0.0001 | 0.0005 |
| PC 32:2 | <0.0001 | <0.0001 |
| PC 32:3 | <0.0001 | 0.9734 |
| PC 32:4 | <0.0001 | 0.3626 |
| PC 32:5 | <0.0001 | 0.6267 |
| PC 33:0 | <0.0001 | 0.6000 |
| PC 33:1 | <0.0001 | <0.0001 |
| PC 33:2 | <0.0001 | 0.1021 |
| PC 33:3 | <0.0001 | 0.4400 |
| PC 33:4 | <0.0001 | 0.0014 |
| PC 33:5 | <0.0001 | <0.0001 |
| PC 34:1 | 0.3527 | 0.0196 |
| PC 34:2 | <0.0001 | <0.0001 |
| PC 34:4 | 0.8884 | 0.0160 |
| PC 34:6 | <0.0001 | <0.0001 |
| PC 35:3 | 0.0001 | 0.0033 |
| PC 35:1 | 0.2309 | <0.0001 |
| PC 35:2 | 0.0002 | 0.0302 |
| PC 35:4 | 0.0004 | <0.0001 |
| PC 35:5 | <0.0001 | 0.0015 |
| PC 35:6 | <0.0001 | 0.0048 |
| PC 36:1 | <0.0001 | 0.2186 |
| PC 36:3 | <0.0001 | 0.0450 |
| PC 36:6 | 0.0029 | <0.0001 |
| PC 36:8 | 0.0004 | <0.0001 |
| PC 37:0 | <0.0001 | 0.1078 |
| PC 37:1 | <0.0001 | 0.0042 |
| PC 37:2 | <0.0001 | <0.0001 |
| PC 37:5 | 0.0096 | 0.0219 |
| PC 38:0 | 0.0001 | 0.0178 |
| PC 38:1 | <0.0001 | <0.0001 |
| PC 38:2 | <0.0001 | <0.0001 |
| PC 38:3 | <0.0001 | 0.0175 |
| PC 38:5 | <0.0001 | <0.0001 |
| PC 38:6 | 0.0007 | 0.4698 |
| PC 39:0 | <0.0001 | <0.0001 |
| PC 39:1 | 0.0001 | 0.4995 |
| PC 39:3 | <0.0001 | 0.4563 |
| PC 39:5 | <0.0001 | <0.0001 |
| PC 40:0 | 0.0419 | 0.0098 |
| PC 40:1 | <0.0001 | <0.0001 |
| PC 40:2 | 0.8399 | <0.0001 |
| PC 40:3 | <0.0001 | <0.0001 |
| PC 40:4 | <0.0001 | 0.0555 |
| PC 40:5 | 0.3577 | 0.0049 |
| PC 41:0 | 0.2411 | 0.0596 |
| PC 41:1 | 0.0016 | <0.0001 |
| PC 41:3 | 0.6566 | <0.0001 |
| PC 42:0 | 0.0058 | 0.0001 |
| PC 42:1 | 0.0054 | <0.0001 |
| PC 42:2 | <0.0001 | <0.0001 |
| PC 42:3 | <0.0001 | <0.0001 |
| PC 42:4 | <0.0001 | <0.0001 |
| PC 42:5 | 0.2490 | <0.0001 |
| PC 43:0 | 0.1549 | 0.0106 |
| PC 43:1 | <0.0001 | <0.0001 |
| PC 43:2 | 0.0001 | <0.0001 |
| PC 44:0 | <0.0001 | 0.0011 |
| PC 44:1 | 0.0005 | 0.0007 |
| PC 44:2 | <0.0001 | 0.0001 |
| PC 44:3 | 0.0156 | 0.9026 |
| PC 44:4 | 0.0040 | 0.0523 |
| PC 46:0 | 0.0052 | 0.1245 |
| PC 46:1 | <0.0001 | <0.0001 |
| PC 46:2 | 0.0026 | 0.0001 |
| PC 46:4 | 0.0959 | 0.0141 |
| SM(d16:1/17:0) | <0.0001 | <0.0001 |
| SM(d16:1/24:1) | <0.0001 | <0.0001 |
| SM(d18:1/19:0) | <0.0001 | <0.0001 |
| SM(d18:1/20:0) | <0.0001 | 0.0032 |
| SM(d18:2/20:1) | 0.0001 | <0.0001 |
| SM(d18:2/21:0) | <0.0001 | <0.0001 |
| SM(d18:2/22:1) | <0.0001 | <0.0001 |
| TG 40:0 | 0.0001 | <0.0001 |
| TG 40:1 | <0.0001 | <0.0001 |
| TG 41:0 | 0.0001 | <0.0001 |
| TG 42:0 | <0.0001 | <0.0001 |
| TG 43:1 | <0.0001 | <0.0001 |
| TG 43:2 | <0.0001 | <0.0001 |
| TG 43:4 | <0.0001 | <0.0001 |
| TG 44:0 | <0.0001 | <0.0001 |
| TG 44:1 | <0.0001 | <0.0001 |
| TG 44:2 | <0.0001 | <0.0001 |
| TG 44:3 | 0.0049 | <0.0001 |
| TG 45:0 | 0.0007 | <0.0001 |
| TG 45:1 | <0.0001 | <0.0001 |
| TG 45:2 | 0.0024 | <0.0001 |
| TG 45:3 | <0.0001 | 0.6993 |
| TG 46:0 | <0.0001 | <0.0001 |
| TG 46:1 | <0.0001 | <0.0001 |
| TG 46:2 | <0.0001 | 0.2629 |
| TG 46:3 | <0.0001 | 0.0002 |
| TG 46:4 | 0.0014 | <0.0001 |
| TG 47:0 | 0.0033 | <0.0001 |
| TG 47:1 | <0.0001 | <0.0001 |
| TG 47:2 | 0.6955 | <0.0001 |
| TG 47:3 | <0.0001 | <0.0001 |
| TG 47:4 | 0.0001 | <0.0001 |
| TG 48:0 | 0.0406 | <0.0001 |
| TG 48:1 | <0.0001 | <0.0001 |
| TG 48:2 | <0.0001 | <0.0001 |
| TG 48:3 | <0.0001 | 0.0002 |
| TG 48:4 | <0.0001 | <0.0001 |
| TG 48:5 | 0.0425 | <0.0001 |
| TG 48:6 | 0.0064 | <0.0001 |
| TG 48:7 | 0.0418 | <0.0001 |
| TG 49:0 | <0.0001 | <0.0001 |
| TG 49:1 | <0.0001 | <0.0001 |
| TG 49:2 | <0.0001 | 0.1784 |
| TG 49:3 | 0.1455 | 0.1562 |
| TG 49:4 | 0.0015 | 0.7719 |
| TG 49:5 | <0.0001 | <0.0001 |
| TG 49:6 | 0.0003 | <0.0001 |
| TG 50:0 | 0.6672 | <0.0001 |
| TG 50:1 | <0.0001 | 0.119 |
| TG 50:2 | <0.0001 | <0.0001 |
| TG 50:3 | <0.0001 | <0.0001 |
| TG 50:4 | <0.0001 | <0.0001 |
| TG 50:5 | <0.0001 | <0.0001 |
| TG 50:6 | <0.0001 | <0.0001 |
| TG 50:7 | 0.2812 | <0.0001 |
| TG 51:0 | <0.0001 | 0.2193 |
| TG 51:1 | 0.0008 | <0.0001 |
| TG 51:2 | <0.0001 | 0.0130 |
| TG 51:3 | 0.0011 | 0.0002 |
| TG 51:4 | <0.0001 | <0.0001 |
| TG 51:5 | 0.1352 | <0.0001 |
| TG 51:6 | <0.0001 | <0.0001 |
| TG 51:7 | <0.0001 | <0.0001 |
| TG 52:0 | 0.0951 | <0.0001 |
| TG 52:1 | <0.0001 | <0.0001 |
| TG 52:2 | <0.0001 | 0.0005 |
| TG 52:3 | <0.0001 | <0.0001 |
| TG 52:4 | <0.0001 | <0.0001 |
| TG 52:6 | <0.0001 | 0.0007 |
| TG 53:0 | <0.0001 | 0.3590 |
| TG 53:1 | <0.0001 | 0.0001 |
| TG 53:2 | <0.0001 | 0.5991 |
| TG 53:3 | <0.0001 | <0.0001 |
| TG 53:6 | <0.0001 | 0.0011 |
| TG 53:7 | 0.0039 | 0.0001 |
| TG 54:0 | 0.0004 | 0.0180 |
| TG 54:1 | 0.0067 | 0.0002 |
| TG 54:2 | <0.0001 | 0.5081 |
| TG 54:3 | 0.1237 | <0.0001 |
| TG 54:4 | 0.0001 | <0.0001 |
| TG 54:6 | 0.0024 | <0.0001 |
| TG 55:0 | 0.0011 | 0.6508 |
| TG 55:1 | 0.0158 | 0.1518 |
| TG 55:2 | 0.1159 | 0.0001 |
| TG 55:3 | <0.0001 | 0.0757 |
| TG 55:4 | 0.0001 | 0.0171 |
| TG 56:0 | <0.0001 | 0.0001 |
| TG 56:1 | 0.0588 | 0.2279 |
| TG 56:2 | <0.0001 | 0.0006 |
| TG 56:3 | 0.7102 | 0.0009 |
| TG 56:4 | 0.7240 | 0.0366 |
| TG 57:0 | 0.0130 | 0.0043 |
| TG 57:1 | 0.0113 | 0.0289 |
| TG 57:2 | 0.0055 | 0.0036 |
| TG 57:3 | <0.0001 | 0.0056 |
| TG 57:4 | 0.0004 | 0.0587 |
| TG 57:5 | 0.0008 | 0.7864 |
| TG 57:6 | 0.9251 | <0.0001 |
| TG 58:0 | 0.068 | 0.0309 |
| TG 58:1 | 0.0017 | 0.0311 |
| TG 58:2 | <0.0001 | 0.1523 |
| TG 58:3 | <0.0001 | <0.0001 |
| TG 58:4 | 0.0003 | 0.0023 |
| TG 58:5 | <0.0001 | 0.1087 |
| TG 59:0 | 0.0737 | <0.0001 |
| TG 59:1 | <0.0001 | <0.0001 |
| TG 59:2 | 0.0211 | 0.1263 |
| TG 59:4 | 0.4642 | 0.0018 |
| TG 59:5 | 0.0012 | 0.0321 |
| TG 59:6 | 0.0033 | 0.7695 |
| TG 60:0 | 0.0151 | 0.0410 |
| TG 60:1 | <0.0001 | <0.0001 |
| TG 60:2 | <0.0001 | 0.0003 |
| TG 60:3 | <0.0001 | 0.0399 |
| TG 60:4 | 0.0001 | <0.0001 |
| TG 61:0 | <0.0001 | <0.0001 |
| TG 61:1 | 0.0390 | <0.0001 |
| TG 61:2 | 0.0184 | 0.0134 |
| TG 61:3 | 0.0322 | 0.0012 |
| TG 61:4 | 0.0116 | 0.6309 |
| TG 61:6 | <0.0001 | 0.0027 |
| TG 62:1 | 0.6000 | <0.0001 |
| TG 62:2 | <0.0001 | <0.0001 |
| TG 62:4 | 0.0002 | 0.3151 |

**Table S2 List of altered metabolites of each mutant strains against the wild type in days 10 of adult worms.** Detailed description showed in the Table S1.
